# Surgical-site infection after hip fracture surgery: preoperative full-body disinfection compared to local disinfection of the surgical site—a population-based observational cohort study

**DOI:** 10.1007/s41999-022-00640-6

**Published:** 2022-04-07

**Authors:** Noelle Probert, Åsa G. Andersson, Anders Magnuson, Elin Kjellberg, Per Wretenberg

**Affiliations:** 1 Faculty of Medicine and Health, Örebro University, Örebro, Sweden; 2 Centre of Clinical Research, Region Värmland, Karlstad, Sweden; 3 Department of Geriatrics, Faculty of Medicine and Health, Örebro University, Örebro, Sweden; 4 Clinical Epidemiology and Biostatistics, School of Medical Sciences, Örebro University, Örebro, Sweden; 5 Department of Infectious Diseases, Central Hospital of Kristianstad, Kristianstad, Sweden; 6 Department of Orthopaedics, Faculty of Medicine and Health, Örebro University, Örebro, Sweden

**Keywords:** Hip fracture, Surgical-site infection, Disinfection, Hip fracture surgery

## Abstract

**Purpose:**

Swedish national guidelines recommend full-body disinfection (FBD) with 4% chlorhexidine before hip fracture surgery to prevent surgical-site infection (SSI) despite little evidence. Our objective was to compare preoperative FBD with local disinfection (LD) of the surgical site regarding SSI incidence.

**Methods:**

All patients with hip fracture, operated at a hospital in Sweden, January 1, 2018 to December 31, 2019 were included. Patients in 2018 (*n* = 237) were prepared with FBD and patients in 2019 (*n* = 259) with LD. Primary outcome was SSI and secondary outcome was SSI and/or death. We adjusted for potential confounders with logistic regression. The adjusted analysis was performed in two models to enable assessment of variables that lacked either outcome; in the first model, these variables were not adjusted, and the second model was restricted to a sub-population not affected by respective variables.

**Results:**

There were 16 (6.8%) cases of SSI in 2018 and 8 (3.1%) cases in 2019. FBD (2018) compared to LD (2019) presented an adjusted OR of 1.9 (95%CI 0.8–4.9, *P* = 0.16) respectively 2.0 (95%CI 0.8–5.1, *P* = 0.14) in the two models of the logistic regression. In addition, 40 (16.9%) patients in 2018 and 29 (11.2%) patients in 2019 had the combined outcome of SSI and/or death, adjusted OR 1.6 (95% CI 0.9–2.8, *P* = 0.08) respectively 1.7 (95% CI 0.9–2.9, *P* = 0.06).

**Conclusion:**

We found a non-significant increased risk of SSI 2018 compared to 2019 after adjustment. Randomized control trials are needed. Nonetheless, results suggest that LD is not inferior to FBD regarding SSI prevention, meaning patients could potentially be spared substantial pain.

## Key summary points

**Aim:**

To compare preoperative full-body disinfection (FBD) prior to hip fracture surgery with local disinfection (LD) of the surgical site regarding incidence of postoperative surgical-site infection (SSI), both procedures performed with 4% chlorhexidine.

**Findings:**

There were 16 (6.8%) cases of SSI in 2018 when FBD was performed and 8 (3.1%) cases in 2019 when LD was performed. FBD (2018) compared to LD (2019) presented an adjusted OR of 2.0 (95% CI 0.8–5.1) in the logistic regression analysis.

**Message:**

Results suggest that LD is not inferior to FBD regarding SSI prevention, meaning patients could potentially be spared significant levels of pain caused by FBD.

## Introduction

Surgical-site infection (SSI) after hip fracture surgery is a disastrous complication associated with increased mortality [1, 2]. SSIs are commonly divided into superficial infection of the skin or subcutaneous tissue and deep infection of the fascia, muscle and prosthetic devices or implant material [3]. Incidence varies from 1 to 8%, deep infection representing 1–2% [1, 2, 4–8]. Numerous risk factors have been identified, both related to patient characteristics [6, 7, 9–11], and to surgery [4–7, 12–14]. Association has also been identified for postoperative factors, such as increased length of stay (LOS), readmission [15], and other infections [16, 17].

The source of pathogens is often the endogenous flora of the patient’s skin and *Staphylococcus aureus (S. aureus)* is the most commonly isolated pathogen [1–3]. Therefore, an obvious strategy for SSI prevention is preoperative skin disinfection. The Swedish Handbook for Healthcare recommends that patients planned for procedures posing a risk of infection by skin-colonizing bacteria go through full-body disinfection (FBD) with 4% chlorhexidine preoperatively. This method is well established and has been recommended for several years due to research presenting evidence [18, 19]. However, according to more recent studies questioning the method, FBD decreases the amount of skin-colonizing bacteria, but it is uncertain whether this results in a reduction of SSIs and systematic reviews present that there in fact does not seem to be any clear evidence of benefit in using FBD with 4% chlorhexidine compared to local disinfection of the surgical site (LD), placebo, no wash or regular soap in terms of SSI prevention [20–24]. Due to the notion of this over the past years, the recommendation is only carried out by approximately 50% of all orthopedic clinics in Sweden [25].

The objective of the study was to compare incidence of SSI between traditional FBD prior to hip fracture surgery with LD of the surgical site, both procedures performed with 4% chlorhexidine.

## Patients and methods

### Study design, setting and participants

In this retrospective population-based observational cohort study, all hospitalizations of patients with acute hip fracture, classified with International Classification of Disease, tenth revision (ICD-10) codes: S72.0 (cervical hip fracture), S72.1 (pertrochanteric hip fracture) or S72.2 (sub-trochanteric hip fracture) who underwent hip fracture surgery at Karlskoga Hospital in Sweden between January 1, 2018 and December 31, 2019 were consecutively included.

### Study intervention

In 2018 preoperative disinfection was performed as FBD with 4% chlorhexidine meaning patients were showered twice during one occasion taking place on a specific shower-gurney. In 2019 preoperative disinfection was performed as LD of the planned surgical site with 4% chlorhexidine meaning patients were disinfected once during one occasion in their own bed. The change in method of disinfection at the orthopedic ward was planned (in line with other orthopedic clinics in Sweden, as mentioned in the introduction) and therefore initially unrelated to this study. During both years, the respective procedures were performed once within 24 h of surgery. If time to surgery was longer than 24 h, disinfection was repeated. All procedures were performed by nursing staff of the orthopedic ward. For each patient, a standardized form was completed addressing how the preoperative washing was performed. If no form was available, information on disinfection was obtained from patient medical records. According to routines of the Orthopedic ward, all patients received antibiotic prophylaxis preoperatively. Patients prepared for arthroplasty received Cloxacillin 2 g×3 at set times preoperatively, patients with penicillin-allergy receiving Clindamycin 600 mg×3. Patients prepared for osteosynthesis obtained Cefuroxime 1.5 g×3, patients with penicillin-allergy receiving Clindamycin 600 mg in a single dose.

### Patient characteristics and confounders

Data were obtained through retrospective review of medical records by use of a standardized review protocol. Initially, patients were observed during hospitalization and all medical records regarding in-patient care within time of follow-up were reviewed. After discharge, medical records regarding in-patient or out-patient care were reviewed for the remaining time of follow-up. Follow-up time was until 6 weeks postoperatively [26].

The following patient information was obtained to characterize the two cohorts: fracture type, length of stay (LOS), pre- and perioperative antibiotics, other infections apart from SSI defined as other antibiotic-treated conditions (not including antibiotic-treated *Clostridium difficile* enterocolitis, cholecystitis caused by gallstones and pyelitis caused by kidney stones), SSI, readmission (into in-patient care) and death. In addition, according to published literature, the following factors identified as significantly associated with SSI were recorded and categorized accordingly: sex [11], age (< 80, ≥ 80 [6], comorbidities [9], American Society of Anesthesiologists classification (ASA class) (≤ 3, > 3) [7], current smoking [7, 9], BMI [6, 7, 9, 10], ongoing anticoagulant therapy [9], ongoing corticosteroid therapy [10], time to surgery (from time of X-ray) (< 24 h, ≥ 24 h) [4], surgical length (< 120, ≥ 120 min) [6, 7], experience of surgeon (less-experienced surgeon or senior surgeon according to working title) [5, 12, 13], reoperation (not related to SSI) [12], and operation with arthroplasty (as opposed to internal fixation) [5, 27].

Comorbidities were collected according to registered ICD-10 codes and comorbidities registered in a standardized form in the surgical records. A Charlson Comorbidity Index (CCI) was calculated according to the coding system by Ludvigsson et al. [28]. Cognitive impairment is a risk factor of SSI [29] and a relevant characterizing factor when it comes to geriatric populations and was therefore presented separately in Table 1, in addition to being included in the CCI calculated for each patient. We defined cognitive impairment as all patients diagnosed with ICD-10 codes of dementia and delirium (F00-F05). The code E11.9 (uncomplicated type 2 diabetes) was the most common code for diabetes among patients in this study but is not included in this coding system for CCI. Therefore, due to that specifically, diabetes mellitus has been identified as an important risk factor of SSI [10], diabetes mellitus was presented independently and therefore not included in the CCI calculated for each patient. SSI was defined as patients diagnosed with ICD-10 codes of superficial infection of the surgical wound or deep infection of prosthetic devices or implant material by a clinician during follow-up. Information on collected microbial cultures and isolated pathogens was also retrieved from medical records.

**Table 1:** Characteristics of patients in cohort 2018 and 2019

Patient characteristics	FBD, 2018 *n* = 237	LD, 2019 *n* = 259	*P*
Age, mean (SD)	81 (10)	83 (10)	0.02
Age > 80, *n* (%)	147 (62)	183 (71)	0.04
Female, *n* (%)	155 (65)	176 (68)	0.55
Type of fracture, *n* (%)			
S72.0—Cervical	133 (56)	115 (44)	0.01
S72.1—Pertrochanteric	86 (36)	117 (45)	0.04
S72.2—Sub-trochanteric	18 (8)	27 (10)	0.27
CCI, mean (SD)	5 (2)	5 (2)	0.39
ASA class ≥ 3	152 (59)	122 (51)	0.11
Cognitive impairment	47 (20)	55 (21)	0.70
Diabetes Mellitus			
All	49 (21)	36 (14)	0.05
Insulin-dependent	23 (10)	16 (6)	0.15
Current smoking	13 (6)	8 (3)	0.19
Anticoagulant therapy	33 (14)	43 (17)	0.41
Corticosteroid therapy	12 (5)	17 (7)	0.48
Hospitalization			
Surgery within 24 h, *n* (%)	155 (65)	186 (72)	0.12
Surgical length, minutes, median (IQR)	70 (51–97)	64 (43–89)	0.02
Less experienced surgeon	17 (7)	19 (7)	0.94
Reoperation (not due to infection)	13 (6)	6 (2)	0.07
Arthroplasty			
All	95 (40)	83 (32)	0.06
Cervical fractures	94 (71)	80 (70)	0.85
Pre-operative antibiotics, *n* (%)	9 (4)	8 (3)	0.67
Peri-operative antibiotics, *n* (%)	215 (91)	237 (92)	0.76
LOS, median (IQR)	6 (4–8)	6 (4–7)	0.42
Readmission	39 (17)	53 (21)	0.25

*FBD* full-body disinfection, *LD* local disinfection, *SD* standard deviation, *CI* confidence interval, *CCI* Charlson Comorbidity Index, *ASA class* American Society of Anesthesiologists Classification system, *IQR* inter-quartile range, *LOS* length of stay

### Primary and secondary outcome measures

Our primary outcome was incidence of SSI, and our secondary outcome was incidence of SSI and/or death. There were patients who died during the 6 weeks follow-up and therefore the secondary outcome was included; due to that, the outcome of SSI within follow-up could not be ruled out in deceased patients.

### Statistical analyses

Differences in age and CCI between the two cohorts were analyzed by independent sample *t* test, differences in LOS and duration of surgery were analyzed by the Mann–Whitney *U* test and differences in categorical variables with the chi-square test.

Unadjusted and adjusted logistic regressions were performed for the SSI and the SSI and/or death outcome to compare the two cohorts. Adjustment was made for the potential confounders presented above under data collection. All variables were evaluated on categorical scale except for CCI evaluated on continuous scale. However, the adjustment could not be performed for smoking and surgeon experience for the SSI outcome and for smoking for the SSI and/or death outcome due to no outcome events among current smokers and/or patients operated by a less-experienced surgeon. Therefore, two adjusted models were performed, the first with no adjustment for the named variables and the second where the adjusted analysis was restricted to the subgroup of non-smoking patients (SSI and/or death outcome) and non-smoking patients operated by a senior surgeon (SSI outcome). The restricted analysis for the SSI outcome included 442 of the 496 (89%) patients. Logistic regression gives odds ratio (OR) with 95% confidence intervals (CI) as association measures. A P value lower than 0.05 was regarded as statistically significant and all analyses were performed in IBM SPSS (Armonk, NY, USA) version 25. A power analysis was performed in retrospect of our study findings, the SSI risk difference (6.8% and 3.1% in the cohorts) and the sample size of 496 patients (237 and 259 in the cohorts) revealing a power of around 50% with the significance level of 5% by the use of chi-square test as statistical method.

## Results

**Fig. 1:**
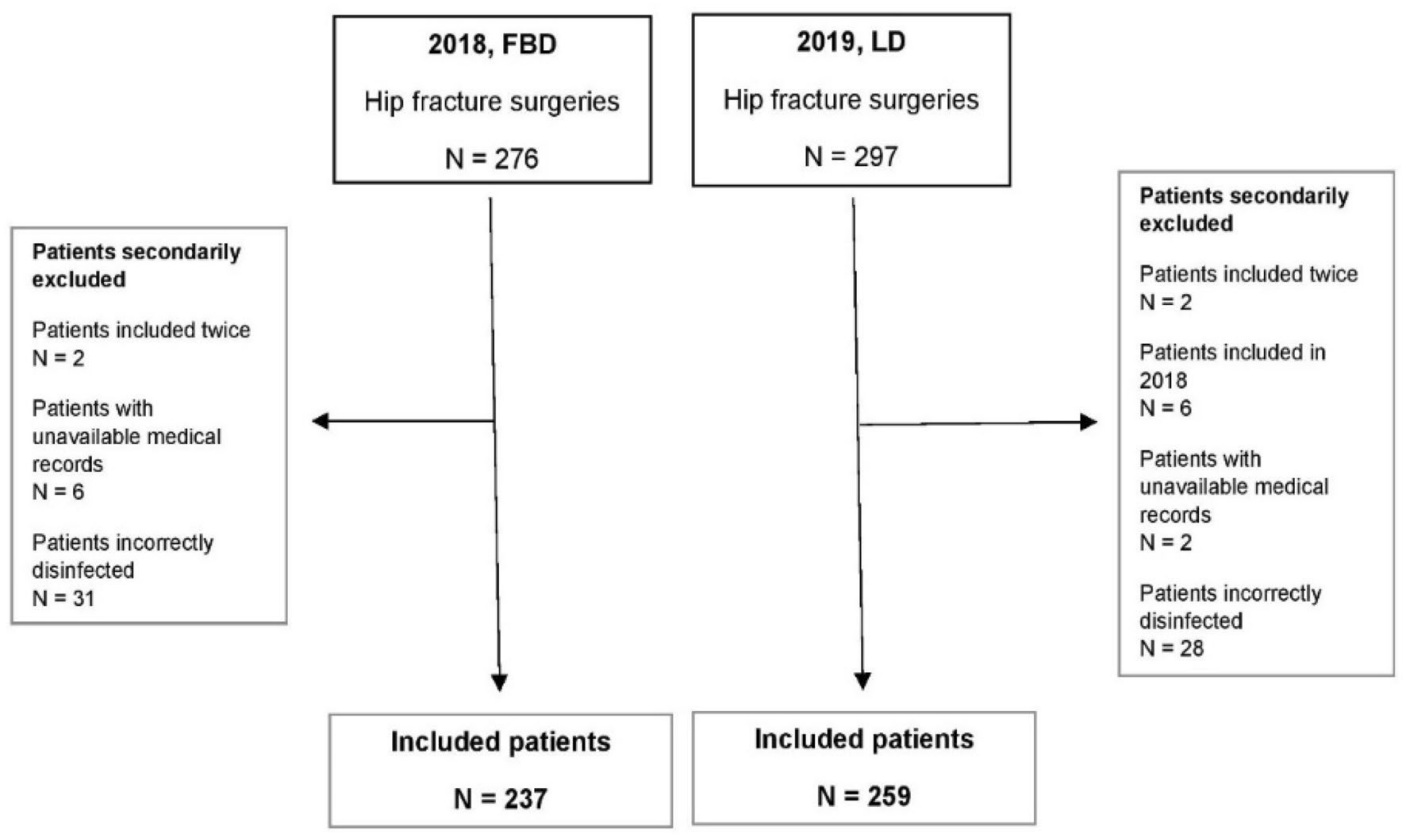
Study design of included patients who went through hip fracture surgery in 2018 and 2019. Abbreviations: *FBD* full-body disinfection, *LD* Local disinfection

As presented in Fig. 1, 237 and 259 hospitalizations were included for further analysis. Hospitalizations of patients with unattainable medical records, of patients who suffered from a second fracture during inclusion and of patients who did not receive disinfection according to correct routine were secondarily excluded.

As seen in Table 1, patients in 2019 had a slightly higher mean age while cervical fractures were significantly more common, and patients had a significantly higher frequency of surgeries < 120 min in 2018. BMI was only found for 12% respectively 6% of patients in 2018 and 2019 and is not included in Table 1.

There were 16 (6.8%) cases of SSI in in 2018 and 8 (3.1%) cases of SSI in in 2019 (Table 2) with an unadjusted OR of 2.3 (95% CI 0.9–5.4, *P* = 0.06) and an adjusted OR of 1.9 (95% CI 0.8–4.9, *P* = 0.16) in the model with no adjustment for smoking and surgeon experience, respectively 2.0 (0.8–5.1, *P* = 0.14) in the population restricted to non-smokers operated by a senior surgeon. In both adjusted models CCI score, reoperation and arthroplasty were associated with a statistically significant increased risk of SSI.

**Table 2:** Unadjusted and adjusted logistic regression for the SSI outcome

	SSI	Unadjusted (*n* = 496)		Adjusted 1^a^ (*n* = 496)		Adjusted 2^b^ (*n* = 442)	
	*n* (%)	OR (95% CI)	*P*	OR (95% CI)	*P*	OR (95% CI)	*P*
FBD, 2018	16 (6.8)	2.3 (0.9–5.4)	0.064	1.9 (0.8–4.9)	0.16	2.0 (0.8–5.1)	0.14
LD, 2019	8 (3.1)	Reference		Reference		Reference	
Age < 80 years	7 (4.2)	Reference		Reference		Reference	
Age ≥ 80 years	17 (5.2)	1.2 (0.5–3.0)	0.65	1.0 (0.4–2.7)	0.98	0.8 (0.3–2.1)	0.60
Male	7 (4.2)	Reference		Reference		Reference	
Female	17 (5.1)	1.2 (0.5–3.0)	0.66	1.4 (0.5–3.7)	0.49	1.3 (0.5–3.4)	0.59
CCI, per unit		1.3 (1.0–1.6)	0.03	1.4 (1.1–1.9)	0.01	1.6 (1.1–2.1)	< 0.01
ASA class ≤ 3	11 (5.0)	Reference		Reference		Reference	
ASA class > 3	13 (4.7)	0.9 (0.4–2.2)	0.96	0.6 (0.2–1.6)	0.29	0.5 (0.2–1.5)	0.22
No DM	18 (4.4)	Reference		Reference		Reference	
DM	6 (7.1)	1.6 (0.6–4.3)	0.30	1.1 (0.4–3.2)	0.84	1.1 (0.4–3.1)	0.92
No current smoker	24 (5.1)	Reference					
Current smoker	0 (0.0)	NE^c^					
No anticoagulant therapy	22 (5.2)	Reference		Reference		Reference	
Anticoagulant therapy	2 (2.6)	0.5 (0.1–2.1)	0.49	0.6 (0.1–3.0)	0.55	0.6 (0.1–2.8)	0.49
No corticosteroid therapy	22 (4.7)	Reference		Reference		Reference	
Corticosteroid therapy	2 (6.7)	1.4 (0.3–6.4)	0.63	1.4 (0.3–7.2)	0.67	1.3 (0.2–6.6)	0.76
Surgery after 24 h	6 (3.9)	Reference		Reference		Reference	
Surgery within 24 h	18 (5.3)	1.4 (0.5–3.6)	0.50	1.7 (0.6–4.7)	0.31	1.8 (0.6–4.9)	0.27
Surgical length < 120 min	22 (4.9)	Reference		Reference		Reference	
Surgical length ≥ 120 min	2 (4.7)	1.0 (0.2–4.2)	0.95	1.0 (0.2–4.7)	0.97	1.1 (0.2–5.0)	0.94
Senior surgeon	24 (5.2)	Reference					
Less experienced surgeon	0 (0.0)	NE^c^					
No reoperation	20 (4.2)	Reference		Reference		Reference	
Reoperation	4 (21.1)	6.1 (1.8–20.0)	< 0.01	7.5 (2.0–27.9)	< 0.01	6.9 (1.8–26.0)	< 0.01
No arthroplasty	9 (2.8)	Reference		Reference		Reference	
Arthroplasty	15 (8.4)	3.2 (1.4–7.4)	< 0.01	3.5 (1.4–8.6)	< 0.01	3.3 (1.3–8.2)	0.01

*SSI* surgical-site infection, *OR* Odds ratio, *CI* confidence interval, *CCI* Charlson Comorbidity Index, *ASA* American Society of Anesthesiologists Classification system, *DM* Diabetes Mellitus

^a^Adjusted 1, Study population was all patients with adjustment for all variables except smoking and patients operated by a less-experienced/senior surgeon due to no SSI outcome

^b^Adjusted 2, Study population restricted to non-smokers and patients operated by a senior surgeon with adjustment for all other variables

^c^NE No estimate due to no SSI outcomes in current smoker and less-experienced surgeon

In addition, 40 (16.9%) patients in 2018 and 29 (11.2%) patients in 2019 had the combined outcome of SSI and/or death (Table 3), with an unadjusted OR of 1.6 (95 CI 0.9–2.7, *P* = 0.07) and adjusted OR of 1.6 (95% CI 0.9–2.8, *P* = 0.08) in the model with no adjustment for smoking, respectively 1.7 (0.9–2.9, *P* = 0.06) in the restricted non-smoking population.

**Table 3:** Unadjusted and adjusted logistic regression for the SSI and/or death outcome

	SSI and/or Death	Unadjusted (*n* = 496)		Adjusted 1^a^ (*n* = 496)		Adjusted 2^b^ (*n* = 475)	
	*n* (%)	OR (95% CI)	*P*	OR (95% CI)	*P*	OR (95% CI)	*P*
FBD, 2018	40 (16.9)	1.6 (0.9–2.7)	0.07	1.6 (0.9–2.8)	0.08	1.7 (0.9–2.9)	0.06
LD, 2019	29 (11.2)	Reference		Reference		Reference	
Age < 80 years	15 (9.0)	Reference		Reference		Reference	
Age ≥ 80 years	54 (16.4)	2.0 (1.1–3.6)	0.03	1.3 (0.7–2.6)	0.41	1.1 (0.6–2.2)	0.76
Male	27 (16.4)	Reference		Reference		Reference	
Female	42 (12.7)	0.74 (0.4–1.3)	0.27	0.8 (0.4–1.4)	0.38	0.8 (0.4–1.3)	0.31
CCI, per unit		1.4 (1.2–1.6)	< 0.01	1.3 (1.1–1.6)	< 0.01	1.4 (1.1–1.7)	< 0.01
ASA class ≤ 3	20 (9.0)	Reference		Reference		Reference	
ASA class > 3	49 (17.9)	2.2 (1.3–3.8)	< 0.01	1.4 (0.7–2.7)	0.29	1.4 (0.7–2.6)	0.36
No DM	55 (13.4)	Reference		Reference		Reference	
DM	14 (16.5)	1.3 (0.7–2.4)	0.46	0.9 (0.5–1.8)	0.80	0.9 (0.4–1.7)	0.66
No current smoker	69 (14.5)	Reference					
Current smoker	0 (0.0)	NE^c^					
No anticoagulant therapy	56 (13.3)	Reference		Reference		Reference	
Anticoagulant therapy	13 (17.1)	1.3 (0.7–2.6)	0.38	0.9(0.4–1.9)	0.83	0.9 (0.4–1.9)	0.80
No corticosteroid therapy	64 (13.7)	Reference		Reference		Reference	
Corticosteroid therapy	5 (16.7)	1.3 (0.5–3.4)	0.65	0.8 (0.3–2.4)	0.73	0.8 (0.3–2.3)	0.67
Surgery after 24 h	27 (17.4)	Reference		Reference		Reference	
Surgery within 24 h	42 (12.3)	0.7 (0.4–1.1)	0.13	0.8 (0.4–1.4)	0.38	0.8 (0.5–1.4)	0.44
Surgical length < 120 min	63 (13.9)	Reference		Reference		Reference	
Surgical length ≥ 120 min	6 (14.0)	1.0 (0.4–2.5)	0.99	1.1 (0.4–2.7)	0.88	1.0 (0.4–2.6)	0.96
Senior surgeon	66 (14.3)	Reference		Reference		Reference	
Less experienced surgeon	3 (8.3)	0.5 (0.2–1.8)	0.32	0.6 (0.2–2.1)	0.42	0.7 (0.2–2.4)	0.55
No reoperation	64 (13.4)	Reference		Reference		Reference	
Reoperation	5 (26.3)	2.3 (0.8–6.6)	0.12	2.1 (0.7–6.4)	0.21	2.0 (0.7–6.3)	0.22
No arthroplasty	37 (11.6)	Reference		Reference		Reference	
Arthroplasty	32 (18.0)	1.7 (0.9–2.8)	0.05	1.6 (0.9–2.8)	0.07	1.7 (0.9–2.9)	0.07

*SSI* surgical-site infection, *OR* Odds ratio, *CI* confidence interval, *CCI* Charlson Comorbidity Index, *ASA* American Society of Anesthesiologists Classification system, *DM* Diabetes Mellitus

^a^Adjusted 1, Study population was all patients with adjustment for all variables except smoking due to no SSI/death outcome

^b^Adjusted 2, Study population restricted to non-smokers with adjustment for all other variables

^c^NE No estimate due to no SSI outcomes in current smoker

**Fig. 2:**
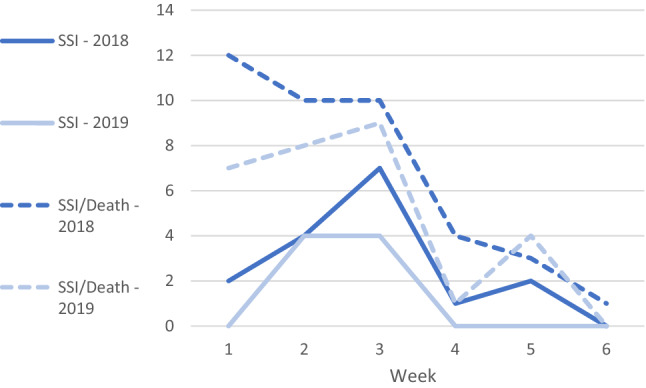
Time of SSI and SSI and/or death during the follow-up time of 6 weeks postoperatively in 2018 and 2019. Abbreviations: *SSI* surgical-site infection

Two cases of SSI in 2018 and one case in 2019 were deep infections of the prosthetic devices or implant material, treated by further surgery. The other cases of SSI were superficial infections of the surgical wound, treated with antibiotics. All SSI diagnoses were based on either clinical symptoms of infection and/or positive microbial culture. Most cases were detected within 3 weeks postoperatively (Fig. 2); 75% and 88% respectively detected after discharge. Re-disinfection due to postponed surgery was performed on 8 (3.4%) patients in 2018 and 4 (1.5%) patients in 2019 (*P* = 0.19), no cases of SSI were detected among these patients.

*S. aureus* was isolated in four cultures in 2018 and one culture in 2019. Other positive cultures presented CoNS, mono-microbial growth of Gram-negative microorganisms or poly-microbial growth. MRSA was not detected in any cultures. In seven of the SSI cases, no culture was taken, and in one case, the culture was negative.

The cohorts did not differ regarding other infections apart from SSI (divided by origin), presenting the following incidences: urinary tract infections with 34 (14.3%) cases in 2018 and 35 (13.5%) cases in 2019 (*P* = 0.79), airway infections with 11 (4.6%) cases in 2018 and 14 (5.4%) cases in 2019 (*P* = 0.70), skin infections with 6 (2.5%) cases in 2018 and 6 (2.3%) cases in 2019 (*P* = 0.88) and infections of unknown origin with 2 (0.8%) cases in 2018 and 2 (0.8%) cases in 2019 (*P* = 0.93).

## Discussion

In this retrospective population-based observational cohort study, the results showed a non-significant difference with an adjusted OR of 2.0 when traditional FBD before hip fracture surgery was compared to LD in terms of SSI incidence. Due to few cases of SSI, the study was somewhat underpowered which prevents us from reaching clearer results. Nonetheless, results indicate that the method of LD does not seem to be inferior to traditional FBD in terms of SSI prevention.

Patients were alike in baseline characteristics (Table 1), values also coinciding with national data [25]. There were significant differences in fracture type between the years although this has not been associated with SSI incidence [1, 5, 7]. Known risk factors of SSI that differed significantly between the cohorts in Table 1 were increased mean age > 80 [6], (although in contradiction patients in 2019 were older) and surgical time of > 120 min [6, 7], higher in 2018. The found incidence of SSI [6, 7], timing of detection (Fig. 2) [30], and isolated pathogens in positive cultures [1, 2, 30], resembles what others have reported.

Our main finding suggesting that the change in method from traditional FBD to LD does not seem to have caused an increased incidence of SSI is in general supported. Bonnevialle et al. compared patients prepared with an antiseptic shower (polyvidone iodine) twice before elective hip replacement with emergency patients not prepared at all and found no cases of SSI in either cohort [23]. Rotter et al. compared FBD with chlorhexidine before clean surgery with a detergent not containing chlorhexidine and found that the relative risk of wound infection in the chlorhexidine group was 1.11% (CI 0.69–1.82) in comparison to the non-chlorhexidine group [21]. Systematic reviews by Webster et al. including all kinds of surgery in addition to Jivegård et al. and Franco et al. addressing all kinds of clean surgery found no evidence of benefit in preoperative FBD with 4% chlorhexidine compared to placebo, soap, and no washing in terms of SSI incidence. However, in contradiction, Wihlborg et al. conducted a study in 1987 similar to ours but reported of a significantly lower rate of SSI in patients preoperatively prepared with 4% chlorhexidine FBD (1.7%) compared to LD of the surgical area (4.1%), RR 0.4 (CI 0.19–0.85), although addressing patients who went through biliary tract, inguinal hernia or breast surgery [31].

The role of chlorhexidine and FBD in SSI prevention seems to be unclear. Although, it remains surprising that even after adjustment for confounders, the cohort prepared with FBD had an odds ratio of 2.0 compared to LD in terms of association with SSI risk. This association has not been recorded to the same extent or not at all in other studies as mentioned above; however, these studies are not directly comparable due to differences, such as included surgeries, type of antiseptic used and diagnostic criteria of SSI, etc. Interestingly, it has been reported by others that disinfection with chlorhexidine prior to hip and knee arthroplasty as well as cardiac surgery does not seem to eradicate bacteria but decreases bacterial diversity [32], and in some cases, increases presence of Gram-negative bacteria, possibly reducing colonization resistance [33]. These findings could potentially explain our results although this is purely speculative. Anyhow, LD does not seem to be inferior to traditional FBD in terms of SSI prevention and if chlorhexidine does in fact have a role in this, LD is a more humane alternative for all patients considering the pain caused by FBD, especially when it comes to frail and potentially cognitively impaired patients, overrepresented within this patient category.

Results of the logistic regression analysis for our primary outcome of SSI compared to the composite outcome of SSI and/or death were similar and we found that increased CCI, reoperation and arthroplasty were significantly associated with SSI risk, in line with others [5, 9, 12, 27]. The two respective models of the adjusted analysis also presented similar results and it is strengthening that the restricted analysis regarding the outcome of SSI does in fact include almost the entire study sample (442 of 496, 89%).

### Limitations and strengths

This study is limited by its retrospective design and that patients were not randomized to receive either method of disinfection. In addition, due to that the cohorts were not compared during the same year, the interventions were not compared during the same time period and the lack of information regarding potential confounders, such as seasonal variability, variances in personnel, etc. is a limitation. The study is also limited by a power of 50% to detect a significant difference which must be considered when interpreting the results. SSIs are multifactorial and while we assessed the potential confounding of the majority of known preoperative risk factors, the risk factors: preoperative serum albumin [6, 7], fasting blood glucose [7], hemoglobin [10], and CRP [14], postoperative use of wound drainage [6], long-term catheterization [34], postoperative hematoma [12], and details regarding method of fracture fixation [5, 11], could not be assessed. BMI (specifically BMI > 28) is an important, independent risk factor of SSI [6, 7, 9, 10], unfortunately BMI was only found for 12% respectively 6% of patients in this study and therefore could not be further assessed. This study is based on medical records, also a limitation due to the risk of inconsistency and error in registration, potentially affecting data and reliability of adjustment for confounders. This limitation is specifically relevant for smoking which was low in our study and potentially underestimated. Finally, our follow-up time of 6 weeks risks missing cases of late chronic wound infection, however, since we wanted to capture SSIs potentially associated with factors of surgery such as preoperative disinfection, a longer follow-up time was considered inaccurate. In addition, other studies have found that the majority of SSIs after hip surgery occur within 4 weeks postoperatively [26]. In terms of strengths, our study is population-based and in line with clinical reality in that almost all eligible patients were included. Contributing factors to this were that written consent was not needed for inclusion, there were no exclusion criteria, and consecutive exclusion was low, this in turn increasing generalizability. In addition, a majority of previously known confounders have been taken into consideration and adjusted for. To our knowledge, this is the first study of its kind in Sweden, addressing a matter potentially causing unnecessary pain for patients. Sweden does represent one of the highest incidences of hip fracture worldwide [35], highlighting the importance of research within the field.

## Conclusion

In conclusion, when comparing traditional FBD with 4% chlorhexidine prior to hip fracture surgery with LD of the surgical site in terms of SSI incidence, we found a non-significant increased risk of SSI in 2018 (FBD) compared to in 2019 (LD) after adjustment. The study has limitations and randomized control trials are needed. Nonetheless, results suggest that LD is not inferior to FBD regarding SSI prevention, meaning patients could potentially be spared significant levels of pain.

## Data Availability

Data are available upon reasonable request. The datasets used and/or analyzed during the current study are available from the corresponding author on reasonable request.
