# Correction to: Surgical-site infection after hip fracture surgery: preoperative full-body disinfection compared to local disinfection of the surgical site—a population-based observational cohort study

**DOI:** 10.1007/s41999-022-00659-9

**Published:** 2022-05-19

**Authors:** Noelle Probert, Åsa G. Andersson, Anders Magnuson, Elin Kjellberg, Per Wretenberg

**Affiliations:** 1 Faculty of Medicine and Health, Örebro University, Örebro, Sweden; 2 Centre of Clinical Research, Region Värmland, Karlstad, Sweden; 3 Department of Geriatrics, Faculty of Medicine and Health, Örebro University, Örebro, Sweden; 4 Clinical Epidemiology and Biostatistics, School of Medical Sciences, Örebro University, Örebro, Sweden; 5 Department of Infectious Diseases, Central Hospital of Kristianstad, Kristianstad, Sweden; 6 Department of Orthopaedics, Faculty of Medicine and Health, Örebro University, Örebro, Sweden

## Correction to: European Geriatric Medicine 10.1007/s41999-022-00640-6

In this article affiliation 1 was missing for authors Åsa G. Andersson and Per Wretenberg. The original article has been corrected.

